# Double auricles of the right atrium: a unique anatomic deformity

**DOI:** 10.1186/1471-2261-11-17

**Published:** 2011-04-19

**Authors:** Georgios I Tagarakis, Dimos Karangelis, Marios E Daskalopoulos, Dimitrios Papadopoulos, Theocharis Koufakis, Ioannis Karantzis, Stefania S Lampoura, Serapheim Chlapoutakis, Nikolaos B Tsilimingas

**Affiliations:** 1 Department of Cardiovascular and Thoracic Surgery, University Hospital of Thessaly, Larissa, Greece; 2 Intensive Care Unit, General Hospital of Larissa, Larissa, Greece

## Abstract

**Background:**

Anatomic deviations, especially those detected during the course of an operation, are medically intriguing, as they raise concerns about their clinical significance and putative complications.

**Case presentation:**

We present, to our knowledge, for the first time a case of an anatomic deviation in the form of a second right atrial auricle in a 70 year-old, coronary bypass-operated male Caucasian patient of Greek origin. No complications were noted intra-or postoperatively.

**Conclusions:**

A second right atrial auricle was found intraoperatively, without causing any clinical complications, or obstructing the normal course of a surgical procedure.

## Background

The auricles of the right and left atrium are prominent anatomic structures of the heart. The anatomic area of the right atrial auricle, which lies in the proximity of the basic rhythm center of the heart, the sinus node, is used for the venous cannulation of the heart before the extracorporeal circulation is entered. To date, no important deformities have been reported in regards to this anatomic formation. The presence of a second atrial auricle, which is presented herein, is to our knowledge, the first reported in the medical literature.

## Case presentation

**Figure 1:**
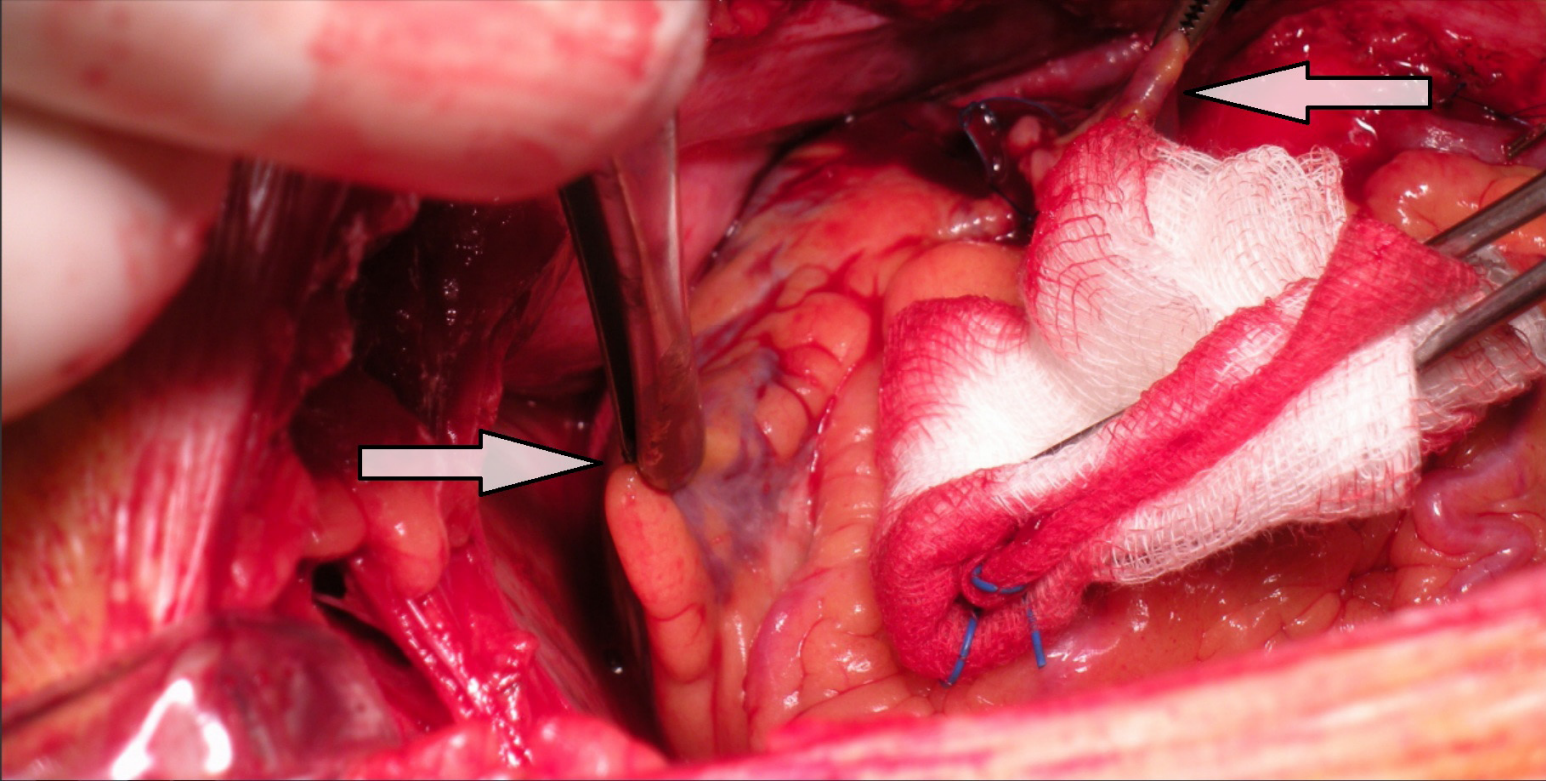
**The two auricles of the right atrium intraoperatively, are held with the surgeon's forceps**. Inferiorly and to the left of the image lies the extra auricle, superiorly and to the right of the image lies the indigenous auricle.

A 70-year old Greek male patient was referred to our department for elective coronary artery bypass surgery, fulfilling the necessary eligibility criteria for this operation. After routine preoperative preparation, the patient was transferred to the operating room, where after the chest and pericardium was opened, a second right atrial auricle was noted. The extra auricle was situated approximately 6 centimeters to the right and inferiorly of the normally placed auricle, and had about the same size and structure with the latter (Figure 1). We could detect no other morphological abnormalities, as for example equivalent deformities of the left atrium or coronary artery disorders, in form of fistulas, aneurysms or course deviations. The operation was conducted in the usual fashion (triple bypass surgery with one internal mammary artery and two saphenous vein grafts), without any complications (e.g arrhythmias) associated with the extra auricle. The patient was discharged on the 9th postoperative day.

## Discussion

**Figure 2:**
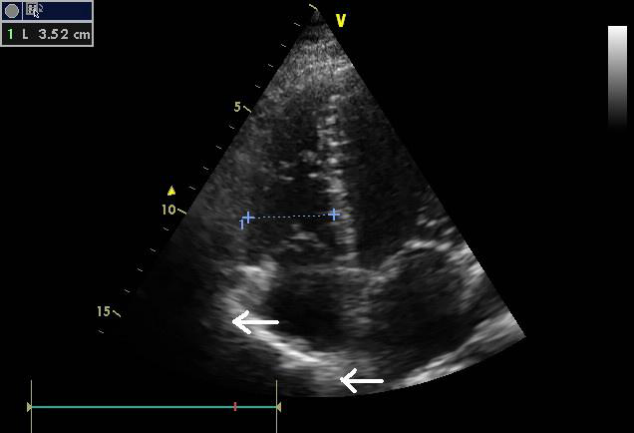
**Postoperative echocardiography image showing the second atrial auricle (superior arrow), as well as the indigenous auricle on the bottom of the image (inferior arrow)**.

To our knowledge, this is the first report of such an anatomic deviation. The incidentally found, second right atrial auricle (Figure 1 left arrow) emerged from the anterior wall of the right atrium approximately 6 centimeters inferiorly of the first auricle (Figure 1 right arrow), while the auricle of the left atrium was lying on its indigenous site. The presented finding is different from a case of right juxtaposition. Juxtaposition of atrial appendages is a side-by-side position of the atrial appendages on one side, left or right, instead of on either side of the great arteries [1]. A thorough postoperative transthoracic and transesophageal echocardiogram confirmed the absence of other cardiac abnormalities, consistent with the preoperative assessment. It is of interest that the preoperative echocardiogram did not report the second auricle, probably due to the lack of clinical suspicion; the examiner was likely focused on other clinically relevant abnormalities, such as myocardial mobility, and ejection fraction. In contrast, the second auricle was detected (with relative difficulty) on the postoperative echocardiogram (both transthoracic and transesophageal), as the examiner was aware of its existence and persisted by examining various angles to better access this anatomic formation (Figure 2). Furthermore, the living brother and the son of the patient underwent echocardiography, but no deformities were noted.

As far as the echocardiography diagnosis is concerned, only extra focus based on an augmented clinical suspicion can lead the examiner to specifically investigate the existence of such a deviation. It is the intraoperative confirmation that will definitely provide the proof for the existence of a second right atrial auricle.

## Conclusions

Based on the aforementioned data, the occurrence of the double auricles of the right atrium did not provoke any clinically relevant complications and was not associated with any other genetic, anatomic, or pathophysiological disorders. Its presence did not hinder neither hemodynamically nor functionally the outcome of the operation. More light will be shed in regards to the etiology and clinical importance of double auricles of the right atrium as similar cases might be presented in the future.

## Consent

Written informed consent was obtained from the patient for publication of this case report and accompanying images. A copy of the written consent is available for review by the Editor-in-Chief of this journal.

## Competing interests

The authors declare that they have no competing interests.

## Authors' contributions

GT was a member of the surgical team and wrote the paper. DK was a member of the attending team. MD and DP helped in the literature review, manuscript writing and correcting. TK and IK helped in image acquisition and manuscript correction. SL checked the paper linguistically. SC helped with literature research and copyediting. NT was the chief surgeon, head of the department and performed the final control of the paper.

All authors have read and approved the final manuscript.

## Pre-publication history

The pre-publication history for this paper can be accessed here:

http://www.biomedcentral.com/1471-2261/11/17/prepub
